# Insights into the elimination of vivax malaria in China

**DOI:** 10.1186/s40249-023-01077-0

**Published:** 2023-03-20

**Authors:** Fang Huang, Li Zhang, Zhi-Gui Xia

**Affiliations:** 1 Shanghai Municipal Center for Disease Control and Prevention, Shanghai, 200336 China; 2 National Institute of Parasitic Diseases, Chinese Center for Disease Control and Prevention (Chinese Center for Tropical Diseases Research), NHC Key Laboratory of Parasite and Vector Biology, WHO Collaborating Center for Tropical Diseases, National Center for International Research on Tropical Diseases, Shanghai, 200025 China

**Keywords:** Malaria, *Plasmodium vivax*, Elimination, China

## Abstract

**Background:**

Malaria is caused by multiple parasitic species of the genus *Plasmodium*. *Plasmodium vivax* is the most geographically widespread and poses challenges in elimination due to its unique biological and epidemiological characteristics. The aim of study was to highlight the practices and experience targeting vivax malaria control and elimination in China.

**Main body:**

*P. vivax* malaria was historically endemic in more than 70% of counties in China, with reported vivax malaria cases as high as 26 million a year. After around 70 years of effort, China was certified as malaria-free in June of 2021. The key insights into China’s vivax malaria control and elimination were offered, including radical cure strategies, comprehensive but adaptive strategies targeting species of *Plasmodium* and *Anopheles*, mass drug administration, and case-/focus-centred surveillance and response systems.

**Conclusion:**

The complete global eradication of *P. vivax* and eventually malaria will be more difficult, and China’s practices and experience could be a valuable reference in this campaign.

**Graphical Abstract:**

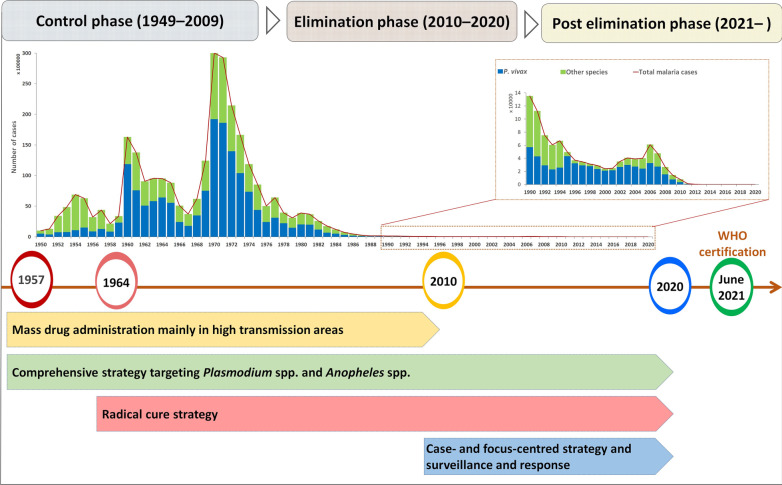

## Background

*Plasmodium vivax*, as the most geographically widespread human malaria parasite, with 4.9 million cases in 2021 globally, has been considered benign for many years [1, 2]. Compared to *P. falciparum*, eliminating *P. vivax* poses different challenges due to its unique biological and epidemiological characteristics, such as persistent hypnozoites in the liver and the current lack of technology to detect them, low parasitaemia in the blood stage impeding diagnosis and treatment, poor treatment adherence for the 14-day course of primaquine (PQ) and its severe side effects in glucose-6-phosphate dehydrogenase (G6PD)-deficient patients, and its wide geographical distribution. Therefore, the most alarming aspect is that the control and elimination of *P. vivax* is more challenging than that of *P. falciparum* [3, 4].

**Fig. 1:**
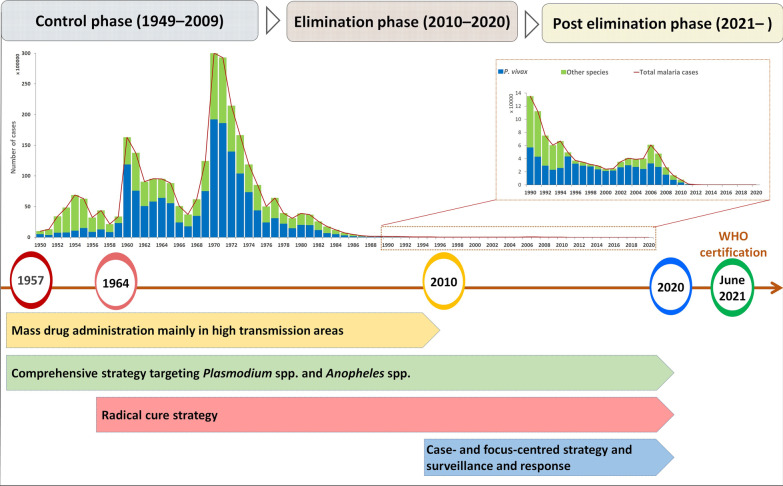
Strategies of *Plasmodium vivax* malaria elimination in different phases in China

In China, four human *Plasmodium* species (*P. vivax*, *P. falciparum*, *P. ovale*, and *P. malaria*e) were historically endemic; among them, *P. vivax* was the most widely distributed in almost all respective areas. It has been estimated that in the 1940s, there were at least 30 million cases of malaria a year in the country, and more than half were vivax malaria infections, which occurred in more than 70% of counties. China experienced two epidemics of vivax malaria in the early 1960s and early 1970s [5]. The main causes were large increasing of mosquito breeding, influx of non-immune populations and interruption of normal malaria control resulted from natural disasters and social and political unrest. Various comprehensive but adapted strategies and interventions were deployed in the following decades according to the characteristics of malaria transmission and vectors in different areas, which included infectious source management, vector control, monitoring and surveillance strengthening, as well as joint prevention and control through regional and multisectoral cooperation. The incidence of vivax malaria declined from 2961.1/100,000 in 1970 to 1.88/100,000 in 2000 [6]. Although a *P. vivax* resurgence and local outbreaks occurred in central China during 2001–2006, this focal endemic was quickly controlled by target interventions, such as mass drug administration (MDA) and case management [7]. In 2010, the Chinese government launched the “National Action Plan for Malaria Elimination (2010–2020)” with the goal of eliminating malaria nationwide by 2020 [8]. China was certified as malaria-free in June of 2021, and the last indigenous case with *P. ovale*, *P. malariae*, *P. falciparum* and *P. vivax* was registered in Guizhou Province in 1962, Hainan Province in 2015, and Yunnan Province in 2015 and 2016, respectively. In the journey of malaria elimination in China, *P. vivax* is the predominant and last malaria parasite eliminated; therefore, several key strategies and interventions targeting vivax malaria control and elimination have been highlighted in this study (Fig. 1).

## Radical cure strategy based on the biological characteristics of *P. vivax* and patient compliance

The main difficulties for *P. vivax* elimination lie in its ability to relapse within weeks to months after the initial infection [9, 10]. Radical cure refers to complete elimination of malaria parasites, which specifically means parasite clearance in both the blood and dormant liver stages. China has a vast territory with many types of climates and different geographical zones. Therefore, the biological characteristics of *P. vivax* in China have different patterns of latency and relapses. Temperate strains of *P. vivax* were mainly distributed in central China with a latent period of hypnozoites that can be as long as 8 to 13 months, while tropical strains occurring in southern China displayed a short latent period. In view of the long treatment course and poor patient adherence to 14-day PQ treatment recommended by the World Health Organization (WHO), a range of clinical trials were undertaken to evaluate the efficacy of different radical treatment regimens and courses against *P. vivax* relapse in central China during the 1960s and 1970s. The most critical differences among the regimens were the total dosage and duration of PQ, such as a total dosage of 100 mg for 5 days, 180 mg for 8 days, 80 mg for 4 days, and 100 mg for 7 days [11]. Nevertheless, the daily dosage of PQ was similar among different regimens, ranging between 22.5 and 30 mg. The 8-day treatment using 180 mg PQ twice for adults with directly observed therapy (DOT) has gradually been recognised after a series of clinical trials and practices, which ensured the patient's medication compliance and the total dosage for clearing the parasites [12]. This regimen was used during illness onset in the transmission season and for anti-relapse treatment in the following spring, the non-malaria transmission season in most parts of China due to the relatively cold climates, to ensure that all the hidden parasites were cleared and no potential source of infection entered the new transmission season from summer to autumn. China had strong three-level primary health care network, and the township and village doctors in collaboration with local governments played a big role in implementing the patient follow-up and spring treatment, providing door-to-door services and DOTs [13]. The guideline for radical treatment of *P. vivax* using this regimen was first formulated in the 1980s [14]. In 1980–1999, a total of 130 million radical treatment regimens were administered nationwide, including 32 million radical treatment regimens, to people who had a history of malaria infection in the previous year. As a result, the malaria burden was reduced by 99.3% in this 20-year period. In 2004, the 8-day PQ radical treatment with DOT was adopted in the national guidelines for malaria treatment to ensure patient adherence. Since 2009, it has been the only regimen recommended by the national guidelines. Although this regimen was different from the WHO guidelines, recommending 14-day PQ treatment [15], it has been proven to be effective in clearing potential parasite reservoirs in the malaria control or elimination phases. Moreover, it increases patient compliance and safety in completing the full treatment.

## Adaptive comprehensive strategy targeting *Plasmodium* spp. and *Anopheles* spp.

China was historically divided into four malaria transmission regions using the 25^th^ and 33^rd^ north parallels as dividing lines in accordance with the geographical characteristics, *Plasmodium* spp. and *Anopheles* spp. as follows: (i) south of the 25^th^ north parallel, (ii) between the 25^th^ and 33^rd^ north parallels, (iii) north of the 33^rd^ north parallel, and (iv) the northwest region. Different targeted interventions were integrated into an adaptive comprehensive strategy based on stratifications and have been adopted since the first national malaria control programme in 1956, which effectively reduced malaria morbidity and mortality in different transmission regions. North of the 33^rd^ north parallels and in the northwest region of China, where *P. vivax* was prevalent with the single vector species of *Anopheles sinensis*, and in the regions between the 25^th^ and 33^rd^ north parallels, where *P. vivax* was predominant (> 74%) and *An. sinensis* was the major vector species [14], the conventional interventions of vector control, such as insecticide-treated bed nets (ITNs) and indoor residual spraying (IRS), would not be the most efficient because *An. sinensis* prefers to biting early in the evening and rest outdoors, and it predominantly uses cattle for blood feeding. The strategy was mostly focused on rapidly detecting, diagnosing, and treating infections through the provision of accessible diagnostic testing and treatment services supplemented with vector surveillance and control. The region south of the 25^th^ north parallel in China contained moderate- to high-transmission areas, where *P. falciparum* and *P. vivax* coexisted, and there was a highly complex situation with the coexistence of multiple vectors and dominance of variable species, such as *An. minimus*, *An. dirus*, and *An. anthropophagus*. *An. minimus* and *An. anthropophagus* are more endophilic, endophagic and anthropophilic mosquitos with higher vector capacity than other *Anopheles* species. Therefore, the comprehensive strategy focusing on vector control, including ITNs and IRS, did play a critical role in reducing the malaria burden in this region, especially in the initial phase of malaria control from 1950 to 1960. In addition, IRS was the key intervention, for foci response, to interrupt transmission and prevent potential transmission in the elimination phase. A total of 5.95 million people in malaria foci were protected by IRS between 2011 and 2020 in the country.

## MDA controlling epidemics and local outbreaks rapidly and effectively

MDA is the administration of full antimalarial treatment to every member of a defined population or every person living in a defined geographical area (except those for whom the medicine is contraindicated) at approximately the same time and often at repeated intervals, which targets the hidden reservoir of malaria parasites. A large-scale MDA with pyrimethamine and PQ was performed and associated with declines in high *P. vivax* transmission in central and southern China [16]. It was estimated that a total of 148.17 million people were administered chloroquine (CQ) and PQ during the spring before the start of the new malaria transmission season, and 202.75 million were given CQ during the transmission season in central China between 1974 and 1986, which substantially decreased the number of malaria cases from 12.8 million in 1973 to 231,000 in 1986 [5]. During 1974–1979, a total of approximately 12 million people were administered MDA in five provinces of central China, where the number of vivax malaria cases rapidly decreased by more than 90% [14]. Targeted MDA in populations staying overnight in the mountains of Hainan Province contributed to the decline in the annual parasite incidence (API) of malaria from 3.5% in 1994 to 0.8% in 1997 [17]. One of the key interventions for immediate response to malaria re-emergence and local outbreaks in Anhui Province in central China included targeting people who lived near water bodies for chemoprophylaxis using 600 mg PQ once a month from August to October in 2007. Therefore, 4.43 million people from 15,740 natural villages in 24 counties of Anhui Province received the treatment, which covered 98.7% of the local population. The incidence of malaria declined by 21.8% in 2007 compared to 2006 [18]. Another major reason for the high efficacy of MDA and targeted MDA in China was the well-organized implementation and adoption of an emergency plan to ensure medication safety and quick responses to the side-effects of PQ [7]. MDA, which was considered for epidemic control as part of the immediate response, might play a significant role in rapidly reducing the incidence of malaria infection and interrupting transmission, and accelerated the progress from control to elimination [7].

## Case- and focus-centred strategy and surveillance and response

China’s surveillance and response system for malaria control and elimination has undergone several major transitions. Between 1950 and 1985, malaria cases were reported and recorded using paper documentation; in 1985–2003, a digital reporting system was gradually developed. In 2004, due to the severe acute respiratory syndrome (SARS) outbreak in China, a novel real-time information reporting management system for infectious diseases, including malaria, was established [19]. After 2010, the national malaria surveillance and response system was updated to transition from the control phase to the elimination phase. Instead of population-based surveillance and response, a case- and focus-centred strategy has been adopted to track the cases, clear-up the focus, and eliminate the infectious sources via the “1-3-7” approach as follows: case reporting within 1 day after diagnosis, case investigation within 3 days and an in-depth focus investigation and response within 7 days. This strategy was targeted to promptly detect and investigate individual malaria infection and ensure appropriate response to interrupt transmission or prevent malaria re-establishment. Meanwhile, the national malaria diagnosis reference laboratory network further ensured that each patient with suspected malaria could be timely and correctly diagnosed and receive the appropriate medication [20]. In 2011, the malaria subsystem was integrated into the web-based National Information Management System for Parasitic Disease Control including the indicators of “1-3-7” approach, which has promoted rapid and accurate interventions and responses. With the effective performance of this strategy in the elimination phase, indigenous *P. vivax* infection was reduced from 677 in 2011 to 24 in 2015. The last indigenous case of *P. vivax* malaria was registered in April of 2016 in the Yunnan Province. China’s “1-3-7” approach could be a tool for malaria elimination in low transmission setting or in the phase moving toward elimination with strong disease surveillance system [21–23].

## Conclusions

In comparison with *P. falciparum*, *P. vivax* is more challenging to eliminate. Through the journey of malaria elimination in China, several adaptive and practical strategies and interventions have been developed and adopted for vivax malaria according to the biological characteristics of local *P. vivax* species. It should be noted that the elimination of any disease is closely related to social-economic development. China made great achievement of economic and social development over the past decades [24], which played important contribution to malaria elimination. The complete global eradication of *P. vivax* and eventually malaria will be more difficult, and China’s practices and experience could serve as a valuable reference in this battle.

## Data Availability

The datasets used and/or analysed during the current study are available from the corresponding author upon reasonable request.

## Abbreviations

API: Annual parasite incidence
CQ: Chloroquine
DOT: Directly observed therapy
G6PD: Glucose-6-phosphate dehydrogenase
IRS: Indoor residual spraying
ITNs: Insecticide-treated bed nets
MDA: Mass drug administration
PQ: Primaquine
SARS: Severe acute respiratory syndrome
WHO: World Health Organization
